# Resolution enhancement of NMR by decoupling with the low-rank Hankel model†

**DOI:** 10.1039/d2cc06682c

**Published:** 2023-04-18

**Authors:** Tianyu Qiu, Amir Jahangiri, Xiao Han, Dmitry Lesovoy, Tatiana Agback, Peter Agback, Adnane Achour, Xiaobo Qu, Vladislav Orekhov

**Affiliations:** a Department of Electronic Science, Fujian Provincial Key Laboratory of Plasma and Magnetic Resonance, Biomedical Intelligent Cloud Research and Development Centre, Xiamen University Xiamen 361005 China quxiaobo@xmu.edu.cn; b Department of Chemistry and Molecular Biology, and Swedish NMR Centre, University of Gothenburg, Box 465 Gothenburg 40530 Sweden Vladislav.Orekhov@nmr.gu.se; c Science for Life Laboratory, Department of Medicine, Karolinska Institute, and Division of Infectious Diseases, Karolinska University Hospital Stockholm 17176 Sweden; d Shemyakin-Ovchinnikov Institute of Bioorganic Chemistry RA Moscow 117997 Russia; e Department of Molecular Sciences, Swedish University of Agricultural Sciences, Box 7015 Uppsala 75007 Sweden

## Abstract

Nuclear magnetic resonance (NMR) spectroscopy has become a formidable tool for biochemistry and medicine. Although *J*-coupling carries essential structural information it may also limit the spectral resolution. Homonuclear decoupling remains a challenging problem. In this work, we introduce a new approach that uses a specific coupling value as prior knowledge, and the Hankel property of the exponential NMR signal to achieve broadband heteronuclear decoupling using the low-rank method. Our results on synthetic and realistic HMQC spectra demonstrate that the proposed method not only effectively enhances resolution by decoupling, but also maintains sensitivity and suppresses spectral artefacts. The approach can be combined with non-uniform sampling, which means that the resolution can be further improved without any extra acquisition time.

## Abstract 2

Virtual broad-band homo-decoupling is demonstrated using Hankel property of the NMR signal. The proposed method enhances resolution and maintains sensitivity.

## Graphical abstract

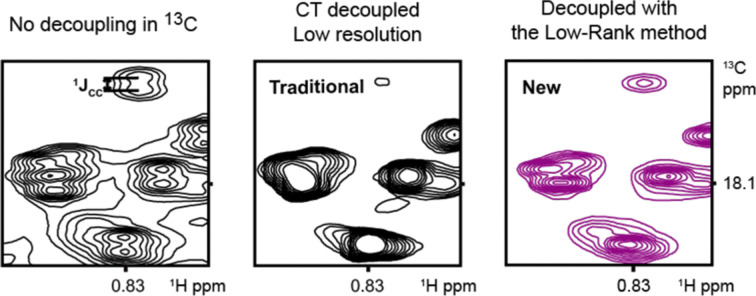

Nuclear magnetic resonance (NMR) spectroscopy is a widely used technique in chemistry,^1^ biology^2^ and medicine.^3^ Resolution enhancement plays an important role in NMR since it determines the quality of the quantitative and qualitative analysis. The improvement of hardware, such as higher magnetic fields, has significantly enhanced resolution.^4,5^ Nevertheless, there are still two main problems that limit spectral resolution.

According to signal processing theory, resolution enhancement requires long acquisition time, *i.e.* more measured data points in the time domain. In multidimensional NMR experiments, this forces the use of very long total measurement time, which is proportional to the number of points for indirect spectral dimensions. However, the appearance of non-uniform sampling (NUS) and reconstruction methods, such as maximum entropy,^6,7^ compressed sensing (CS),^8^ multi-dimensional decomposition (MDD),^9,10^ low-rank Hankel method (LR) and more recently deep learning-based techniques,^11–13^ have greatly alleviated this problem.

The homo-nuclear *J*-coupling causes signal splitting and thus represents another reason for line-broadening and loss of resolution. The decoupling can be achieved in several ways, including the use of the pure shift approach,^14,15^ constant time evolution,^16,17^ bilinear rotational decoupling,^18,19^*etc.*

The mechanism of the *J*-coupling is well understood and the coupling values are known.^20,21^ This information can be exploited to perform decoupling by software deconvolution also known as virtual decoupling (VD).^11,13,22–24^ Decoupling and reconstruction of spectrum from NUS data can be therefore combined and solved by one single method. Furthermore, it was noted that VD is likely to improve NUS reconstruction, because it reduces the number of individual peaks in the spectrum, which can have related implications for different reconstruction algorithms. Thus, in compressed sensing,^8^ VD increases sparseness of the spectrum. Similarly, the low-rank (LR) reconstruction,^25–28^ which is based on the low-rank Hankel property of the time domain free induction decay (FID) NMR signal, benefits from the VD, because the splitting caused by *J*-coupling increases the number of peaks and consequently the rank. This requires an increase of NUS levels or even, when the Hankel matrix is not low-rank anymore, may corrupt the reconstructed spectrum.

In this work, we used a specific coupling value as prior knowledge so that the FID can be reconstructed and decoupled simultaneously. Since the decoupling reduces the number of peaks in the spectrum, the NUS fraction can be further decreased.

A continuous FID signal is modelled as:^28–30^1
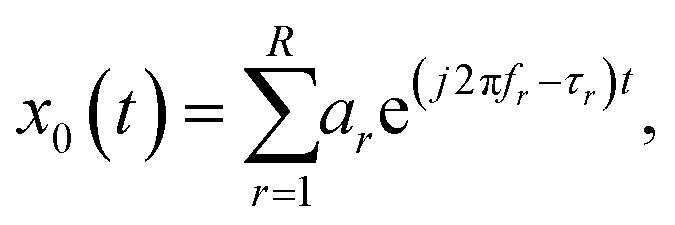
where *a*_*r*_, *f*_*r*_ and *τ*_*r*_ denote the complex amplitude, the central frequency and the damping factor, respectively, and the summation goes over all *R* peaks in the spectrum.

For a *J*-coupled two-spin systems, the measured FID signal is written as:

2 *x*_c_(*t*) = *x*_0_(*t*)*c*(*t*)

where *c*(*t*) = cos π*Jt*^24^ and *J* represents the coupling value.

The proposed low-rank decoupling (LRD) method, thus, aims to recover *x*_0_(*t*) signal from *x*_c_(*t*). The new algorithm is derived from the traditional low-rank Hankel method (LR), which is used for reconstructing spectra from non-uniformly sampled (NUS) data^25,30^ and spectra denoising.^28^ Without specific consideration of *J*-coupling, the LR the spectrum reconstruction is obtained by solving the following optimization problem:3
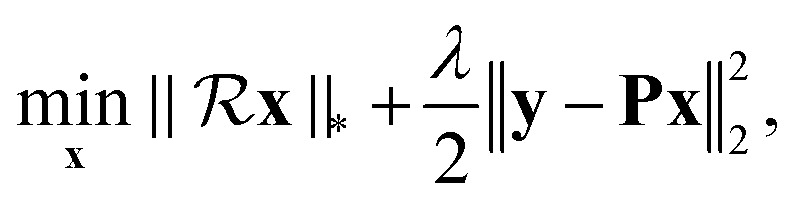
where vector **x** stands for the discrete reconstructed FID signal. Vector **y** represents the measurement in the time domain. Operator 
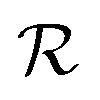
 transforms a vector into a Hankel matrix. *λ* is the regularization parameter. **P** is an identity matrix or, in the case of NUS, it represents a NUS schedule. ‖·‖_*_ and ‖·‖_2_ denote nuclear norm and vector l_2_ norm, respectively. The main idea of the LR method is to minimize the rank of Hankel matrix given by **x**, *i.e.*, the number of exponential components of **x**. The Relationship between a Hankel matrix given by an FID signal and the spectral peaks is illustrated in Fig. S1 in the ESI.†

The low-rank decoupling (LRD) method proposed in this work is defined as:4
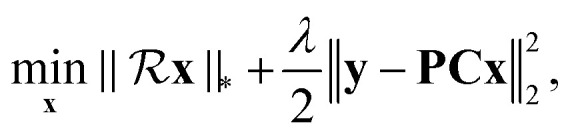
where we introduce matrix **C** defined as diag(**c**). Here c denotes the finite discrete form of *c*(*t*), which is determined by a specific *J*-coupling value considered as a parameter in the method. The algorithm for solving the minimization problem in eqn (4) is given in the ESI.† When the decoupled time domain signal x is recovered, the spectrum is produced by the traditional Fourier transform.

We used simulated and experimental spectra to verify the performance of the LRD methodology. The one-bond couplings occur between adjacent ^13^C atoms, *e.g.* in proteins *C*_*α*_–*C*_*β*_ backbone pairs or between methyl carbons, and their adjacent carbons. It should also be noted that we assumed within the frame of the present study (*i.e.* in eqn (3) and (4)) the typical value for these coupling values as^1^*J*_CC_ = 35 Hz.^31^

The proposed method was compared with conventional decoupling using the iteratively reweighted least square algorithm for compressed sensing (CS-IRLS)^24^ algorithm implemented in the mddnmr software.^32^ The compared method utilizes the same assumption about the decoupling value, but constrains the sparsity in Fourier spectra. For experimental signals, a spectrum decoupled by constant time (CT) evolution sequence, which is very commonly used in most applications,^16^ has been added for comparison.

To ensure a fair comparison, merely the decoupling of the fully-sampled spectra is presented in Fig. 2. The decoupling of 40% NUS signals is presented in the ESI,† illustrating the clear possibility to combine the LRD with NUS for improving of resolution and/or reducing acquisition time.

The results presented in Fig. 1 display a comparison between LRD and CS on a synthetic spectrum. Both methods successfully decouple the spectrum as shown in Fig. 1(b). While CS-IRLS offers a spectrum with a perfect baseline, it also over-sharpens the resonances. Furthermore, it may also weaken the low intensity peaks (note for example the peak marked by arrow in Fig. 1(c)). In contrast, the LRD method performs well, preserving the intensity and providing a comparatively better line shape (as marked by arrows in Fig. 1(d)).

**Fig. 1:**
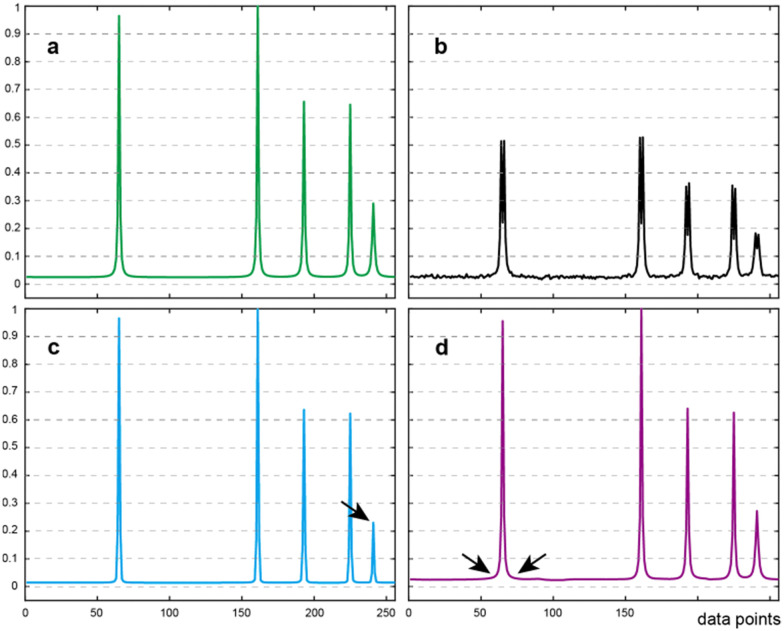
The virtual decoupling in a synthetic spectrum containing five peaks. (a) Is the reference fully-sampled spectrum without *J*-coupling. (b) The spectrum with *J* = 35 Hz. (c and d) Are decoupled spectra by CS-IRLS and by the LRD method proposed within the present study, respectively. Arrows points to the peaks mentioned in the text.

In this part, a 2D HMQC spectrum of 44kDa fragment of the mucosa-associated lymphoma translocation protein 1 (MALT1[Casp-IgL3]_338–719_) is used as an example.^33^ The details of all the performed experiments are presented in the ESI† section.

Fig. 2 displays different decoupling schemes in 2D ^1^H–^13^C HMQC spectrum of MALT1. Although all three tested methodologies decouple the spectrum successfully, clearly noticeable differences in resolution, sensitivity and artefacts can be identified.

**Fig. 2:**
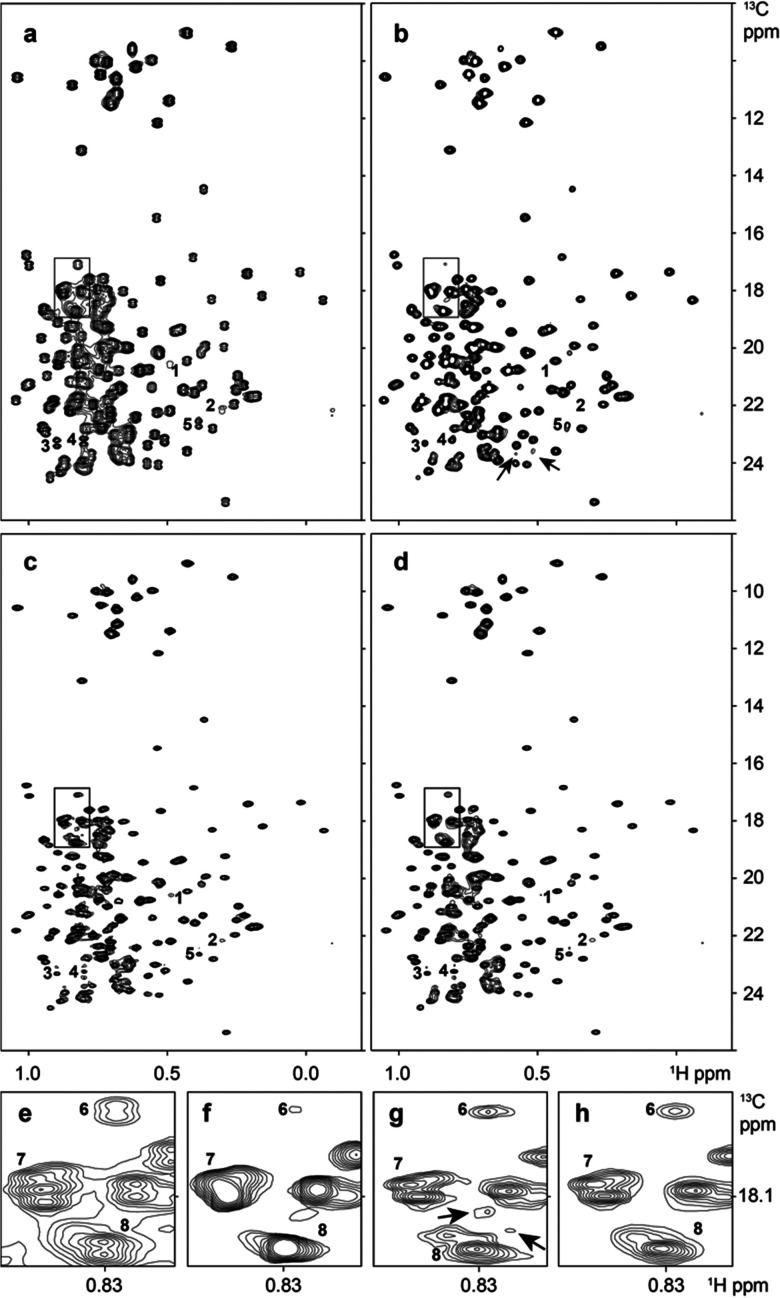
Decoupling of a 2D ^1^H–^13^C HMQC spectrum of MALT1. (a) Is the *J*-coupled spectrum. (b) Is the spectrum obtained using the CT evolution sequence. (c and d) Are virtually decoupled by CS-IRLS and by the proposed method LRD, respectively. Regions marked by black rectangles in (a–d) are enlarged in (e–h), respectively. Peaks and spectral artefacts discussed in the text are indicated by numbers and black arrows, respectively.

Compared to the *J*-coupled spectrum (Fig. 2a), the constant time (CT) evolution offers a spectrum (Fig. 2b) with higher resolution. However, CT may also result in significant sensitivity loss. Indeed, some peaks are clearly weakened, such as peak 6 (Fig. 2(e–h)). Peaks with low intensity (such as peaks 1 and 2) even disappear (Fig. 2(e–h)). This is also clear in 1D cross-sections of peaks 1, 2, and 6 presented in ESI† Fig. S2. Both the CS-IRLS and LRD methods decoupled spectra show better resolution than their CT counterpart due to longer acquisition time allowed by these two VD approaches. As a result, several resolved individual peaks emerged as for example peak groups 7 and 8. These peaks were not discernible in the spectrum decoupled by constant time evolution. In CT experiments, the resolution can be improved only to the expense of further significant loss of sensitivity.

For both CS-IRLS and LRD, some artefacts such as peaks 3, 4 and 5, are caused by significant deviations of the actual coupling from 35 Hz value used for reconstruction. The effect of small deviations of the actual coupling from the value assumed for the reconstruction is illustrated for simulated data in ESI† Fig. S5. The problem can be alleviated in some applications. For example, in HNCA experiments, coupling variations are usually small.^24^ However, as shown in Fig. 2(g), there are some other unignorable artefacts in the CS-IRLS spectrum marked by black arrows. The proposed LRD method seemingly provides a cleaner spectrum, which helps to avoid ambiguity in quantitative and qualitative analyses.

As a note, the model in eqn (2) implies that the target coupling is removed only once, while other couplings regardless of their *J*-coupling values are not affected. For example, a triplet peak with the target coupling value is converted to a doublet.

We present here a new decoupling methodology, named LRD, which is based on the low-rank Hankel model. The one-bond coupling between adjacent ^13^C atoms was taken as an example for the validation of our approach and for comparison with other already established methods. The obtained decoupling results, on both synthetic and experimental spectra, demonstrate that the LRD method is capable of the decoupling, offering higher resolution and significantly cleaner spectra. The presented approach provides a new tool for broadband homonuclear decoupling.

Proposed model and designed numerical experiments: T. Qiu, V. Orekhov. Performed numerical experiment: T. Qiu, A. Jahangiri, and D. Lesovoy. Analysed data: T. Qiu, V. Orekhov. Contributed samples: X. Han, T. Agback, P. Agback, A. Achour. Wrote the paper: T. Qiu, V. Orekhov, X. Qu.

This work was supported, in part, by the National Natural Science Foundation of China (grants 61971361 and 62122064), Xiamen University Nanqiang Outstanding Talents Program, and Chinese Scholarship Council; The Swedish Foundation for Strategic Research grant ITM17-0218 to T. A. and P. A., grant RSF 23-44-10021 to D. L., Swedish Cancer Society grant 21 1605 Pj01H to A. A., the Swedish Research Council grants 2021-05061 to A. A. and 2019-03561 to V. O., and the Swedish Foundation for International Cooperation in Research and Higher Education (STINT) grant CH2017-7231. This study used NMRbox: National Center for Biomolecular NMR Data Processing and Analysis, a Biomedical Technology Research Resource (BTRR), which is supported by NIH grant P41GM111135 (NIGMS).

## Conflicts of interest

There are no conflicts to declare.

## Supplementary Material

CC-059-D2CC06682C-s001
