# Supplementary material for: Resolution enhancement of NMR by decoupling with the low-rank Hankel model
Source: Chem Commun (Camb). 2023 Apr 18;59(36):5475–8. doi: 10.1039/d2cc06682c (PMC10152455; doi:10.1039/d2cc06682c)
Supplement: CC-059-D2CC06682C-s001 [file CC-059-D2CC06682C-s001.pdf]

Electronic Supplementary Material (ESI) for ChemComm.
This journal is © The Royal Society of Chemistry 2023

Supplementary Information

The Low-Rank Hankel reconstruction method

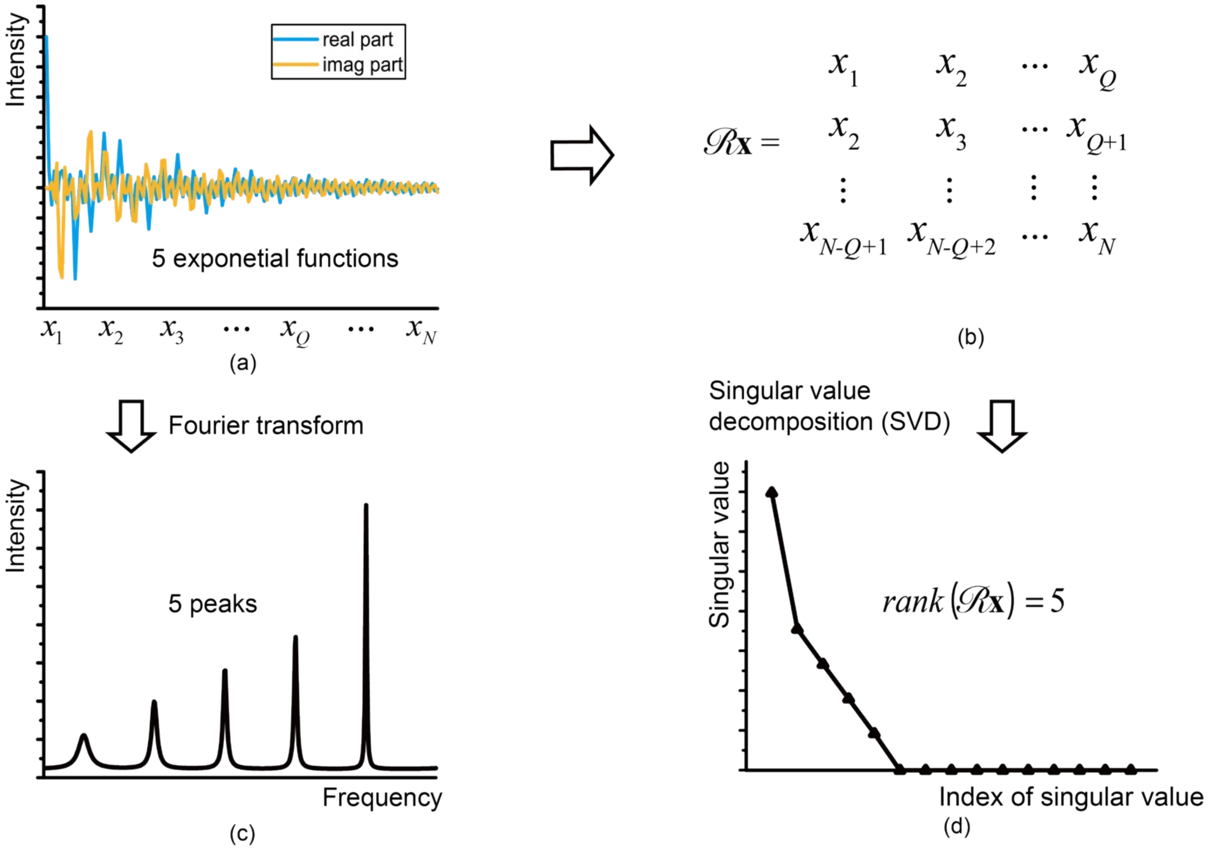

Fig. S1. **Relationship between a Hankel matrix given by a signal in time domain (FID) and the number of exponential components.** **(a)** FID signal ***x*** with blue and yellow curves illustrating it's real and imaginary parts. **(b)** structure of the Hankel matrix obtained from the signal in (a). **(c)** the Fourier spectrum of the signal in (a). **(d)** the singular values of the Hankel matrix in (b).

Algorithm

The model of the proposed LRD method is defined as:

$$\min_{x} \|Rx\|_* + \frac{\lambda}{2}\|y - PCx\|_2^2, \#(A1)$$

where vector $x \in C^{N \times 1}$ stands for the variable that needs to be determined. Vector $y \in C^{M \times 1}$ is the measurement data with coupling. Matrix C is defined as $diag(c)$ which denotes the finite discrete form of Eq. (2) presented in the main text of the manuscript. Operator R transforms a vector into a Hankel matrix. Matrix $P \in R^{M \times N}$ (M $\leq$ N) represents the NUS schedule. Symbol $\|\cdot\|_*$ denotes the nuclear norm defined as the sum of singular values. The regularization parameter λ balances the nuclear norm and consistency.

By introducing two variables Z and D, the augmented Lagrangian formulation of Eq. (A1) is written as:

$$\min_{x,Z} \max_{D} \|Z\|_* + \frac{\beta}{2}\|Rx - Z\|_F^2 + \langle D, Rx - Z \rangle + \frac{\lambda}{2}\|y - PCx\|_2^2, \#(A2)$$

where $\langle \cdot , \cdot \rangle$ denotes the inner product in complex matrices. It is defined as $\langle A,B \rangle = R(tr(\overline{A}B))$, where $\overline{A}$ represents the conjugation of **A**, and the symbol R denotes the real part[1].

Using alternating direction method of multipliers (ADMM)[2], the problem in Eq. (A2) is divided into the following three sub-problems:

$$\begin{cases} \min_x \frac{\beta}{2}\left\|Rx - Z + \frac{D}{\beta}\right\|_F^2 + \frac{\lambda}{2}\|y - PCx\|_2^2 \\ \min_Z \|Z\|_* + \frac{\beta}{2}\left\|Rx - Z + \frac{D}{\beta}\right\|_F^2 \\ D \leftarrow D + \tau(Rx - Z) \end{cases}. \#(A3)$$

The solution to Eq. (A3) is expressed as:

$$\begin{cases} x_{k+1} = (\beta R^* R + \lambda C^H P^H P C)^{-1}\left[\beta R^*\left(Z_k - \frac{D_k}{\beta}\right) + \lambda C^H P^H y\right] \\ Z_{k+1} = S_{1/\beta}\left(Rx_k + \frac{D_k}{\beta}\right) \\ D_{k+1} = D_k + \tau(Rx_{k+1} - Z_{k+1}) \end{cases}, \#(A4)$$

where the subscripts k and k+1 denote the iteration steps. The superscripts H and * denote conjugation transposed and adjoint operator, respectively. β and τ are two parameters. $S_{1/\beta}$ is a singular thresholding operator defined as $S_{1/\beta}(X) = U diag(\{\sigma_r - 1/\beta\}_+)V^H$, where matrix **X** is with singular value decomposition $X = U diag(\{\sigma_r\}_{r=1}^{R})V^H$ and $t_+ = max(0,t)$[3]. R^* denotes an operator that transforms a matrix into a vector by summarizing each skew diagonal. R^*R is defined as an operator satisfying $R^*R\,x = Wx$, where W is a diagonal matrix whose main diagonal is the number of times that an element of x appears in a Hankel matrix[4].

The whole algorithm has been summarized as pseudo code in Table S1.

**Table S1. Pseudo code of the LRD algorithm**
**Input:** y, C, P, λ; **Output**: $\hat{x}$
**Initialization:** $k = 1$, $\beta = 1$, $\tau = 1$, $k_{max} = 2000$, $x_1 = y$
1) **While** $k < k_{max}$ and $\|x_{k+1} - x_k\|_2/\|x_k\|_2 > 10^{-5}$, **do**<br>2) $x_{k+1} = (\beta R^* R + \lambda C^H P^H P C)^{-1}[\beta R^*(Z_k - D_k/\beta) + \lambda C^H P^H y]$;<br>3) $Z_{k+1} = S_{1/\beta}(Rx_k + D_k/\beta)$;<br>4) $D_{k+1} = D_k + \tau(Rx_{k+1} - Z_{k+1})$;<br>5) $k \leftarrow k + 1$;<br>6) **End while**
**Output:** $\hat{x} = x_{k+1}$

The Matlab code used in the work can be found at Github.

https://github.com/TyQiu2/Low-Rank-Hankel-Decoupling

Experiment for the 2D ^{1}H- ^{13}C HMQC spectrum

The sample used for the results presented in Fig. 2 was prepared as described before[5].

Fully-sampled methyl 2D ^{1}H-^{13}C HMQC with 200 complex points in the ^{13}C dimension (46.5 ms acquisition time) was acquired at 298K on a 900 MHz Bruker AVANCE III-HD spectrometer equipped with 3mm cryo-TCI probe. The directly detected dimension of the region of the full reference 2D spectrum (from 1.1 to -0.8 ^{1}H ppm) was processed using the NMRPipe software[6], and imported in MATLAB and qMDD[7] for consecutive reconstruction and decoupling by LRD or CS-IRLS[8]. The NUS schedule satisfying Poisson gap[9] with 40% is generated along the ^{13}C dimension. The parameter of Poisson distribution satisfies a sine-weighted function, changing as the location of sampled points.

The constant-time 2D ^{1}H-^{13}C CT-HMQC (presented in Fig. 2(b) in the main text) with 102 complex points in the ^{13}C dimension (22.5 ms acquisition time) was acquired at 298K on a 900 MHz Bruker AVANCE III-HD spectrometer equipped with a 3mm cryo-TCI probe.

1D traces of peaks in the 2D ^{1}H- ^{13}C HMQC spectrum

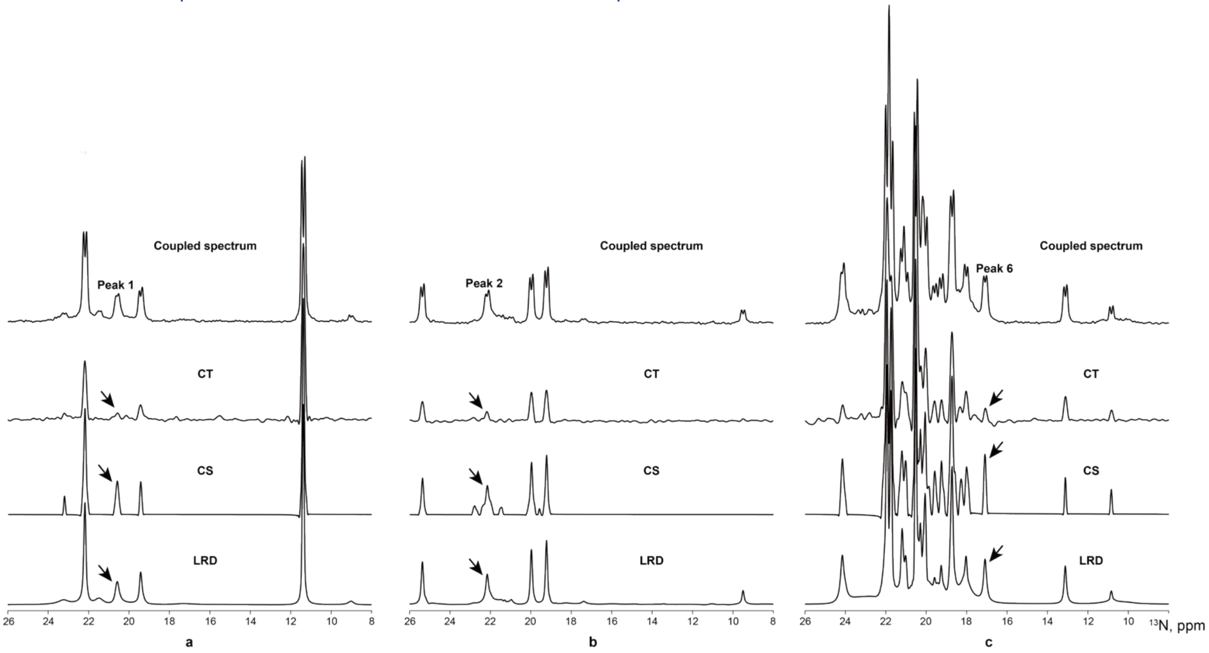

Fig. S2. 1D traces of through peaks 1, 2, and 6 in the 2D ^{1}H-^{13}C HMQC spectra shown in Fig. 2 in the main text. Compared with CS and LRD, the CT decoupling scheme clearly weakens the intensities of peaks 1,2, and 6.

Decoupling from NUS data

The results of our study demonstrate that the proposed LRD method is capable of successfully decoupling fully-sampled spectrums. It is well established that NUS provides a reliable way to enhance resolution. Here, we introduced NUS to our model and verified the effects of this combination on synthetic and experimental spectra, demonstrating that the proposed methodology can significantly improve resolution without any extra acquisition time.

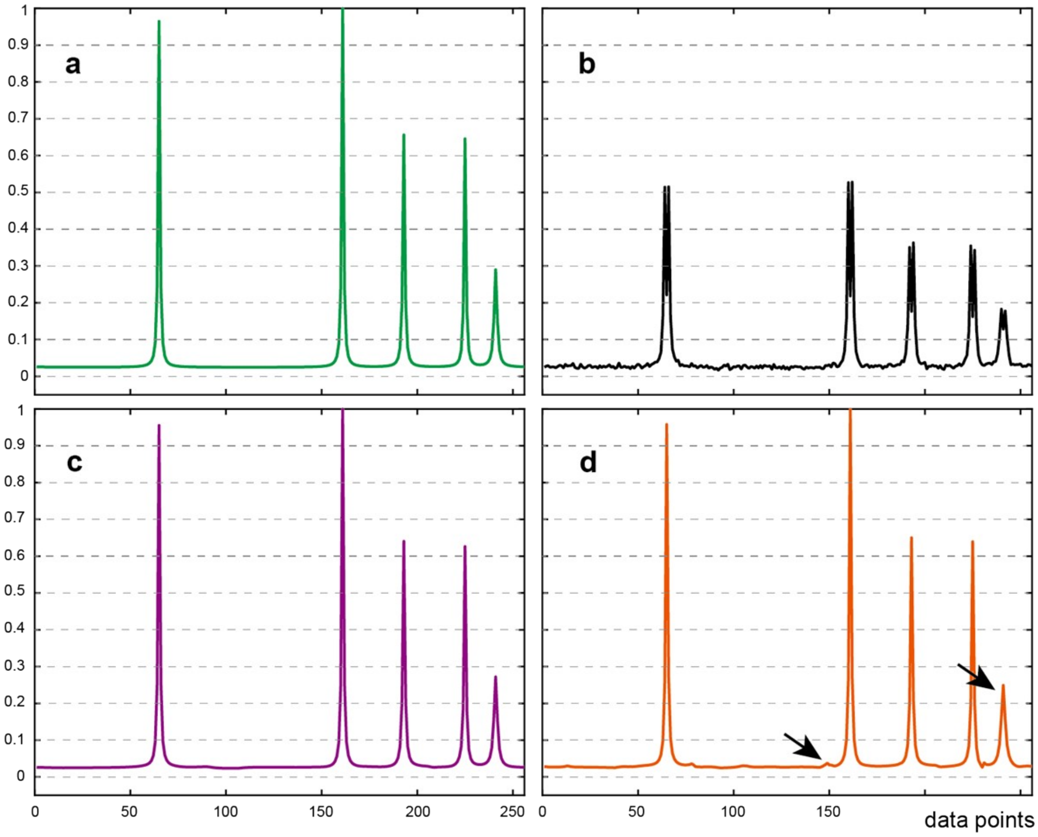

Fig. S3. Decoupled synthetic spectra obtained by using the method presented within the present work. **(a)** denotes the fully-sampled reference spectrum. **(b)** stands for the J-coupled spectrum with J=35 Hz. (**c**) and **(d)** are decoupled spectra from fully-sampled and 25% NUS data, respectively. Black arrows indicate low intensity artefacts at the baseline and the partly weakened lowest peak. It should be noted that a 1D NUS schedule satisfying the Poisson gap[9] was used. The standard deviation of the Gaussian noise in (b) was 0.005.

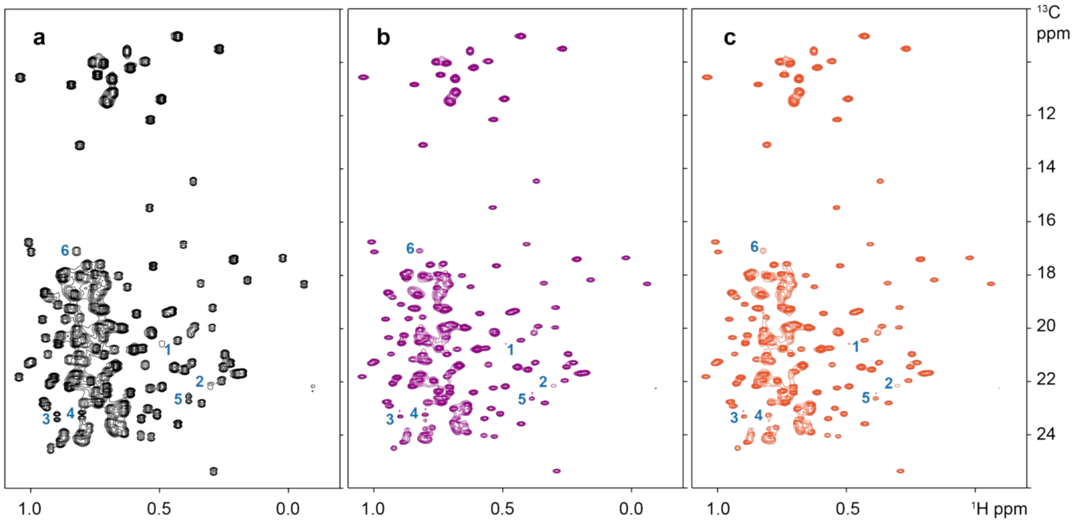

Fig. S4. Decoupling of the 2D ^{1}H-^{13}C HMQC spectrum of MALT1 by by the LRD method. **(a)** is the J-coupled spectrum. **(b)** and **(c)** are decoupled spectra from fully-sampled and 40% NUS, respectively. Although some moderate-intensity peaks are weakened in **(c)** (see for example peak 6), peaks with low intensity such as peaks 1 and 2, are preserved.

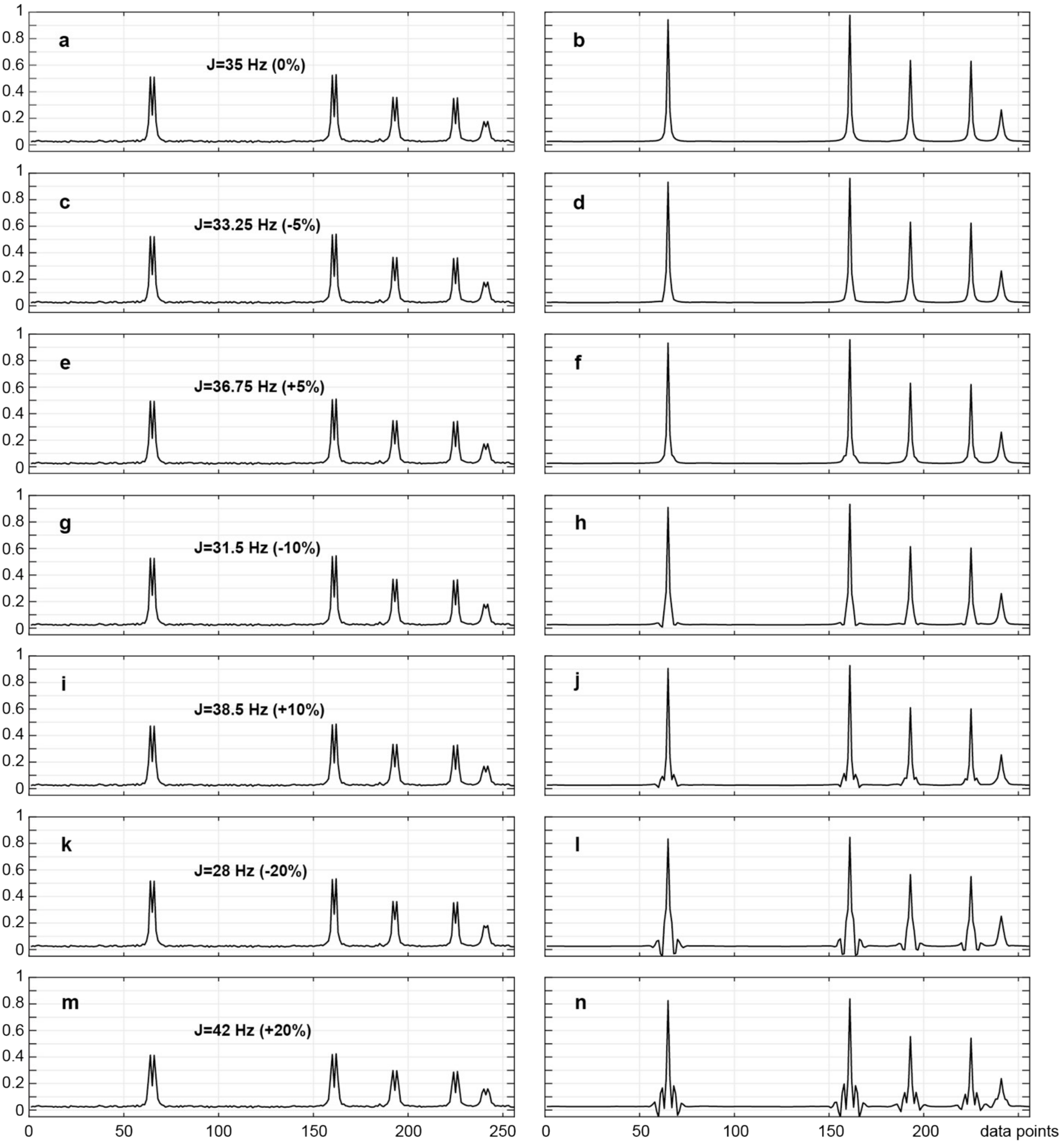

Fig. S5. The effect of small deviations of the J-coupling value in the synthetic spectra from the parameter used in the LRD. The left and right columns depict the coupled and corresponding decoupled spectra, respectively. The numbers in figure show J-coupling values in the spectra (and relative difference from 35 Hz). The proposed method is capable of reasonable decoupling if the J-coupling parameter in the LRD matches the actual coupling in the spectrum within 10%. For the larger discrepancy, significant line-shape artefacts are observed.

References

1 Lu, H. *et al.* Low rank enhanced matrix recovery of hybrid time and frequency data in fast magnetic resonance spectroscopy. *IEEE Transactions on Biomedical Engineering* **65**, 809-820 (2017).

2 Boyd, S., Parikh, N., Chu, E., Peleato, B. & Eckstein, J. Distributed optimization and statistical learning via the alternating direction method of multipliers. *Foundations and Trends® in Machine learning* **3**, 1-122 (2011).

3 Cai, J.-F., Candès, E. J. & Shen, Z. A singular value thresholding algorithm for matrix completion. *SIAM Journal on optimization* **20**, 1956-1982 (2010).

4 Ying, J. *et al.* Vandermonde factorization of Hankel matrix for complex exponential signal recovery—application in fast NMR spectroscopy. *IEEE Transactions on Signal Processing* **66**, 5520-5533 (2018).

5 Unnerståle, S. *et al.* Backbone assignment of the MALT1 paracaspase by solution NMR. *Plos one* **11**, e0146496 (2016).

6 Delaglio, F. *et al.* NMRPipe: a multidimensional spectral processing system based on UNIX pipes. *Journal of biomolecular NMR* **6**, 277-293 (1995).

7 Orekhov, V. Y. & Jaravine, V. A. Analysis of non-uniformly sampled spectra with multi-dimensional decomposition. *Progress in nuclear magnetic resonance spectroscopy* **59**, 271-292 (2011).

8 Kazimierczuk, K. *et al.* Resolution enhancement in NMR spectra by deconvolution with compressed sensing reconstruction. *Chemical Communications* **56**, 14585-14588 (2020).

9 Hyberts, S. G., Takeuchi, K. & Wagner, G. Poisson-gap sampling and forward maximum entropy reconstruction for enhancing the resolution and sensitivity of protein NMR data. *Journal of the American Chemical Society* **132**, 2145-2147 (2010).
